# Peter Chester Chadwick

**Published:** 1903-09

**Authors:** 


### Obituary Notices.

### PETER CHESTER CHADWICK,

One of the oldest practitioners in the West of England, and one who was widely known and much respected, was born in June, 1811, and died in June of this year: he was accordingly

92 years of age, and had been a qualified practitioner for the long period of 68 years. He was the son of J. W. Chadwick, solicitor, of Long Ashton, was educated at Taunton College School under Dr. Follett, father of the late Rector of Winscombe. He was apprenticed at the age of 16 to Messrs. Flower and Leach, of Chilcompton, with whom he worked for five years before commencing his student's course at St. Bartholomew's Hospital. After receiving his diplomas, he became House Surgeon at his hospital for a period of eighteen months: afterwards he practised for a very short time at Portishead, and then in 1837 he commenced his life's work at Wrington. This he carried out with unremitting zeal for a period of fifty years, and the only holiday he ever took was his wedding tour.

Few of his contemporaries survive, but his most notable characteristics will be remembered by those who do: he was a good Latin scholar, and was devoted to his work. He was neat, methodical and orderly in all his arrangements of work and time: his iron constitution and his abstemious life enabled him to manifest untiring industry, and so he won the devotion of several generations of patients who were born with his assistance and who died under his observation. His fondness of children, an inherited Irishman's love for a good horse, and his ability to use plain and emphatic language when occasion required, are qualities which to some extent account for his great popularity with the working classes and the poor.

He had a great regard for his college friends, Sir James Paget and Sir George Burrows, and he used to tell a good story of the early uses of potassic iodide: "We will give the patient the new drug," said the doctor, but "a little blue pill as well."

In his later years he was still devoted to hunting pursuits, and many members of the Stanton Drew and Wells Harriers showed their regard for him by attending his funeral. He was a widower, and left one daughter, the wife of the Rev. Noel Lucas, Rector of Dolberrow, Somerset.

